# The Added Diagnostic Value of Transcatheter CT Hepatic Arteriography for Intraprocedural Detection of Previously Unknown Colorectal Liver Metastases During Percutaneous Ablation and Impact on the Definitive Treatment Plan

**DOI:** 10.1007/s00270-023-03508-9

**Published:** 2023-07-25

**Authors:** Susan van der Lei, Jip Opperman, Madelon Dijkstra, Nikita Kors, Rianne Boon, Bente A. T. van den Bemd, Florentine E. F. Timmer, Irene M. G. C. Nota, Janneke E. van den Bergh, Jan J. J. de Vries, Hester J. Scheffer, Bart Geboers, Timothy Neuss, Evelien Schouten, Birgit I. Lissenberg-Witte, Robbert S. Puijk, Martijn R. Meijerink

**Affiliations:** 1 Department of Radiology and Nuclear Medicine, Amsterdam University Medical Centers, VU Medical Center Amsterdam, Cancer Center Amsterdam, De Boelelaan 1117, 1081 HV Amsterdam, The Netherlands; 2 Cancer Center Amsterdam, Amsterdam, The Netherlands; 3 Department of Epidemiology and Data Science, Amsterdam University Medical Centers, VU Medical Center Amsterdam, Amsterdam, The Netherlands; 4 NWZ Group, Alkmaar, The Netherlands; 5 OLVG Hospital, Amsterdam, The Netherlands

**Keywords:** Colorectal liver metastases (CRLM), Thermal ablation, Radiofrequency ablation (RFA), Microwave ablation (MWA), Transcatheter CT hepatic arteriography (CTHA)

## Abstract

**Purpose:**

This study assessed the diagnostic value of CT hepatic arteriography (CTHA) for the intraprocedural detection of previously unknown colorectal liver metastases (CRLM) and the impact on the definitive treatment plan.

**Materials and Methods:**

All patients treated with CTHA-guided percutaneous ablation for CRLM between January 2012 and March 2022 were identified from the Amsterdam Colorectal Liver Met Registry (AmCORE). Radiology reports of the ablative procedure and follow-up imaging were reviewed to see if (a) previously unknown CRLM were detected intra-procedurally and if (b) new CRLM, potentially missed on CTHA, appeared within 6 months following the procedure; three abdominal radiologists re-reviewed the baseline CTHA scans of these patients with early recurrence. To ratify immediate ablations of concomitantly detected CRLM, the upper limit of false positives was predefined at 10%.

**Results:**

One hundred and fifty-two patients were included. With CTHA, a total of 17 additional tumours in 15 patients were diagnosed and treated immediately, two representing disappeared tumours following systemic chemotherapy. Compared to the conventional contrast-enhanced (ce)CT, ceMRI and 18F-FDG PET-CT, adding CTHA was superior for the detection of CRLM (*P* < .001). Within 12 months of follow-up 121, new CRLM appeared in 49/152 patients (32.2%); retrospective blinded assessment revealed 56 to already be visible on the baseline CTHA scan (46%); four lesions without substrate on follow-up scans were considered false positives (*n* = 4/60; 7%). Arterial ring enhancement was the most frequently reported imaging characteristic (*n* = 45/60; 75%).

**Conclusion:**

The subsequent use of CTHA has added value for the detection of previously unknown and vanished CRLM. Taking into account the low number of false positives (7%) and the favourable safety profile of percutaneous ablation, we believe that immediate ablation of typical ring-enhancing supplementary tumours is justified and sufficiently validated.

**Level of Evidence:**

Level 3; individual cross-sectional study with consistently applied reference standard and blinding.

## Introduction

Colorectal cancer (CRC) is a frequently diagnosed malignancy and the second leading cause of cancer-related mortality in the world. Approximately half of the patients with CRC develop colorectal liver metastases (CRLM) [1–5]. Currently, multiple curative-intent local treatment options are available for patients with liver only or liver dominant disease. Over the past decades, thermal ablation has acquired an important role, either as an adjunct or as a less invasive alternative to partial hepatectomy [4–8].

In conventional computed tomography (CT)-guided liver tumour ablation, a baseline intravenous contrast-enhanced (ce)CT, often including the arterial and portal venous phase, is acquired for treatment planning. Probe placement is usually performed under unenhanced CT fluoroscopy while focusing on unsteady anatomical landmarks or using stereotactic navigation. To timely detect complications and assess technical success, a second ceCT with intravenous contrast is used after the ablation. Confirmation of the ablation zone, if performed at all, is either based on so-called ‘eye-balling’ to subjectively estimate if the tumour-free margin around the initial tumour was achieved or using image registration and confirmation software [9–12].

The transcatheter CT hepatic arteriography (CTHA) technique [9, 12, 13] is a relatively new technique to assist percutaneous thermal ablation procedures in the treatment of hepatocellular carcinoma (HCC), colorectal and non-colorectal liver metastases. This technique entails the selective placement of a catheter in the common or proper hepatic artery, to enable the repeated admission of small doses of intra-arterial intrahepatic iodine-based contrast agent. Compared to conventional ceCT fluoroscopy guidance, CTHA is hypothesized to optimize (a) pre-procedural planning, by (repeatedly) clearly depicting most liver tumours and surrounding blood vessels, (b) intraprocedural targeting by improving tumour conspicuity and (c) image registration and ablation confirmation [11–14]. In case of insufficient tumour coverage by the ablation zone, the needle can be directly repositioned to allow for additional overlapping ablations. CTHA is also known for its ability to differentiate between viable residual tumour tissue and ablative scar tissue (‘incomplete ring sign’), thereby improving intraprocedural monitoring [10–12, 15]. Besides leading to a decrease in the number of patients with local tumour progression (LTP), potentially reducing the number of repeat procedures, CTHA is also thought to visualize metastases at an earlier stage, hence potentially contributing as a diagnostic tool [9–12]. However, the detection of concomitant, previously unknown liver tumours, does not automatically justify immediate local treatment in the same session, as the rate of lesions representing benign liver tumours or heterogeneous perfusion deficits is unknown.

The primary aim of this study was to determine the added diagnostic value of CTHA for the intraprocedural detection of previously unknown CRLM during percutaneous ablation and the impact on the treatment plan. By defining specific imaging characteristics, we intend to validate criteria that help decide whether to ablate immediately or a wait-and-see.

## Materials and Methods

### Study Design and Patient Selection

This study is a retrospective cohort study from a prospectively maintained database (Amsterdam Colorectal Liver Met Registry—AmCORE) and was conducted at a tertiary referral institution for hepatobiliary and gastrointestinal malignancies. The AmCORE database consists of patients with CRLM and contains specific patient-, disease-, tumour- and procedure-related characteristics at baseline and during follow-up. The Institutional Review Board pre-approved the AmCORE registry (reference number 2021.0121) and waived the need for additional medical ethical approval for this specific project. All patients consented to the registration and the catheter-guided percutaneous tumour ablation.

Patients treated with CTHA-guided percutaneous ablation for new CRLM between January 2012 and March 2022 were identified from the prospective database. Supplementary descriptive data were collected from an electronic patient database. Included patients were treated with thermal ablation according to national guidelines, e.g. at least one small unresectable CRLM < 3 cm. A maximum of 6 weeks between the last pre-procedural diagnostic scan(s) and the CTHA-guided procedure was allowed. Routine pre-operative imaging consisted of guideline protocolled ceCT plus ceMRI including diffusion-weighted imaging; the use of 18F-FDG PET-CT was not standardized.

Exclusion criteria were patients aged under 18 years, missing follow-up or follow-up less than 6 months. Patients in whom the catheter was not selectively located in the hepatic artery (because selective placement was not possible or because the catheter was displaced during transport) were noted but excluded from the assessment. The CTHA technique has been described more extensively in the previous publications [9, 12].

All procedure and radiology reports were reviewed by two researchers (MD, 1st year PhD candidate and MD, 4th year resident interventional radiology) to assess whether concomitant, previously unknown, CRLM were found intra-procedurally and if so, whether this impacted the original treatment plan. Patients with rapid disease progression, defined as > 20% growth in the longest diameter of the known CRLM, were noted but excluded from further analysis, assuming that potentially detected concomitant lesions could also be explained by growth over the detection-threshold in between the pre-procedural imaging and the CTHA-guided ablation.

In addition to the analysis of the intraprocedural detected tumours, CTHA was compared to each individual diagnostic modality for the detected of the pre-procedural known tumours.

Patients in whom new CRLM were detected within 12 months on follow-up ceCT, 18F-FDG PET-CT and/or ceMRI were extracted from the database for retrospective review. Routine follow-up imaging post-ablation was 3–4 monthly CEA and 18F-FDG PET-CT and/or ceMRI scans in the first 2 years. Patients with diffuse scattered new CRLM, which makes it impossible to correlate with the initial CTHA, were excluded. The CTHA series were assessed independently, by three academic abdominal radiologists with, respectively, 3, 10 and 14 years of experience, to determine whether the ‘new’ CRLM diagnosed on follow-up imaging were retrospectively visible on CTHA, before (blinded inspection) and after revealing (targeted inspection) the segment and location where the CRLM would later appear. For tumours detected on baseline CTHA, specific characteristics such as overall attenuation compared to surrounding liver parenchyma, conspicuity, delineation and ring enhancement were reported. All radiologists were simultaneously instructed on how to assess the scans in order to minimize interobserver variability.

### Outcome Measure

The primary purpose of this study was to determine the diagnostic value of CTHA (for the intraprocedural detection of previously unknown CRLM) and thereby validating (or rebutting) the immediate ablation of concomitantly detected tumours on CTHA (change of treatment plan). The added diagnostic value of CTHA over conventional ceCT, ceMRI (routine pre-procedural imaging) and, whenever available, 18F-FDG PET-CT (mostly used as problem solver) was defined as the proportion of supplementary detected CRLM. To determine whether immediate ablation for a previously unknown tumour should be preferred over a watch-and-wait approach, the upper limit of false-positive lesions, defined as retrospectively detected on CTHA by radiology review, but without substrate on follow-up imaging, was predefined at 10%.

Secondary endpoints were overall technical success and specifically for the concomitantly treated CRLM, overall complications and complications surely or potentially related to the ablation of concomitantly detected CRLM and, for the retrospective analysis, specific imaging characteristics of newly detected tumours and interobserver variation.

### Statistical Analyses

All statistical analyses were performed using SPSS v. 28 (IBM Corp., Armonk, NY). On account of the dichotomous variables, McNemar tests were used to determine the accuracy in diagnosing new CRLM amongst the different diagnostic tests. Only descriptive statistics such as reporting numbers (with or without percentage), median (interquartile range) or mean (standard deviation) were used. P < 0.05 was considered statistically significant. Interobserver agreement for the retrospective detection of CRLM was assessed using kappa statistics (agreement was considered fair, substantial and excellent for kappa values 0.41–0.6, 0.61–0.8 and 0.81–1, respectively).

## Results

**Fig. 1:**
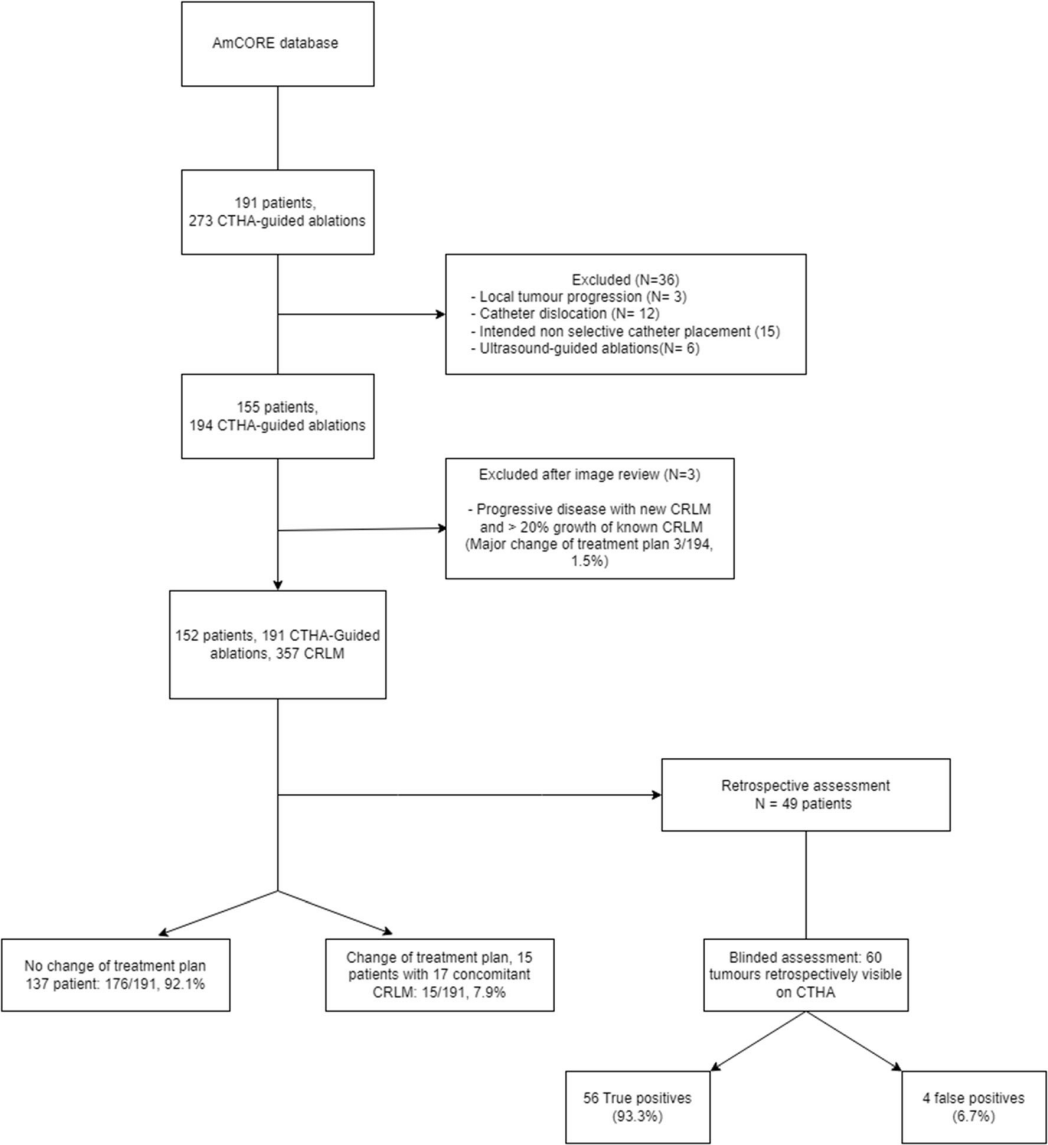
Flowchart of the included patients

Between January 2012 and March 2022, 191 patients treated with 273 transcatheter CTHA-guided percutaneous ablation procedures were assessed. Twenty-seven patients were excluded due to intended non-selective placement (n = 15) or luxation of the catheter during transport (n = 12). After reviewing all procedure records, 155 patients treated with 194 CTHA-guided ablations met the inclusion criteria (Fig. 1, Table 1).

**Table 1:** Patient-, tumour- and procedure-related characteristics

Baseline characteristics			
Patient characteristics	Overall	Concomitant CRLM immediately ablated	Tumours retrospectively detected
Patients (N)	N = 152	N = 15	N = 49
Age, Y, mean (SD)*	65.0 (12.8)	64.7 (16.4)	63.4 (13.0)
Sex, M:F	66%: 34%	67%: 33%	75%: 25%
BMI, mean (SD)*	26.4 (5.0)	26.0 (5.0)	26.6 (0.8)
ASA, mean	2.2	2.2	2.1
Disease characteristics			
*Primary tumour location, N (%)*	34 (22.4)	3 (20.0)	5 (10.2)
Rectum	80 (52.6)	7 (46.7)	31 (63.3)
Colon left-sided and colon right-sided	37 (24.3)	5 (33.3)	13 (26.5)
Tumour characteristics			
Size, mm, Mean (SD)*	17.03 (10.47)	11.12 (6.6)	17.47 (10.6)
Location (Couinaud segment), N (%)			
I	8 (2.2)	1 (5.9)	2 (3.2)
II	30 (8.4)	1 (5.9)	7 (11.3)
III	14 (3.9)	0	1 (1.6)
IVa	38 (10.6)	0	7 (11.3)
IVb	5 (1.4)	1 (5.9)	2 (3.2)
V	43 (12.0)	1 (5.9)	6 (9.7)
VI	50 (14.0)	3 (17.6)	7 (11.3)
VII	82 (23.0)	5 (29.4)	17 (27.4)
VIII	87 (24.4)	5 (29.4)	12 (19.4)
Procedure characteristics			
Induction chemotherapy, N_patients_ (%)	46 (30.3%)	6 (40%)	–
Procedures (N)	191	15	62
*Anaesthesia technique*			
General anaesthesia, N (%)	41 (22)	3 (20)	12 (19)
Sedation (Midazolam), N (%)	27 (14)	3 (20)	8 (13)
Sedation (Propofol), N (%)	117 (61)	9 (60)	40 (65)
*Ablation technique*			
RF ablation N (%)	35 (18)	2(13)	37 (19)
MW ablation N (%)	156 (82)	13 (87)	159 (80)

* = continuous variables reported as mean (standard deviation; SD), BMI = Body Mass Index, ASA = American Society of Anesthesiologists score, RF = Radiofrequency and MW = Microwave

In eighteen procedures (in 18 distinct patients) concomitant, previously unknown, CRLM were detected (n = 18/194; 9.3%). Three procedures were discontinued (n = 3/194; 1.5%), because multiple new CRLM were detected alongside rapid tumour growth of the known CRLM (increase in longest diameter of the known CRLM > 20%), excluding the procedure from further analysis in this study. Eventually, 152 patients underwent 191 CTHA-guided percutaneous thermal ablations (Fig. 1, Table 1). Forty-six patients were treated with induction chemotherapy prior to the procedure (30.3%).

### Previously Unknown Tumours

**Fig. 2:**
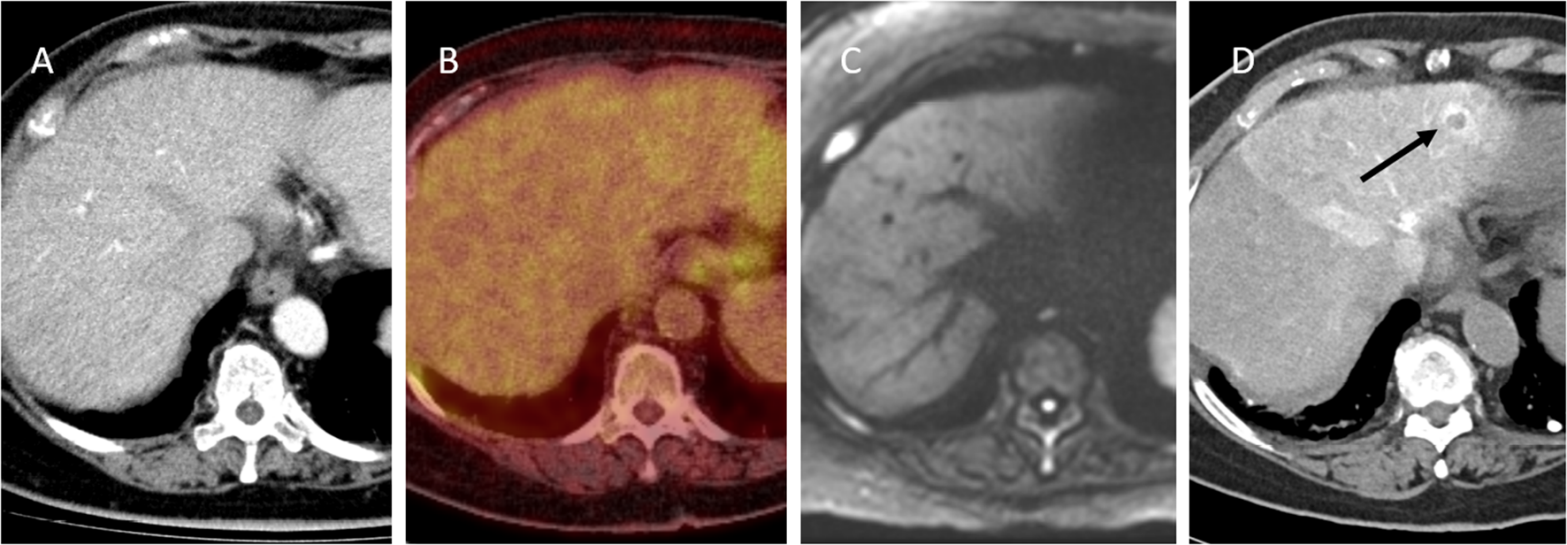
Case with concomitant CRLM:** A** and** B**; pre-procedural contrast-enhanced CT and 18F-FDG PET-CT.** C**: pre-procedural MRI (DWI).** D**: transcatheter CTHA in arterial phase. An additional ring-enhancing lesion in segment II (arrow) was found intra-procedurally. This lesion was considered highly suspect for CRLM and consequently ablated in the same procedure. This lesion was not detected with the conventional pre-procedural diagnostic modalities

**Fig. 3:**
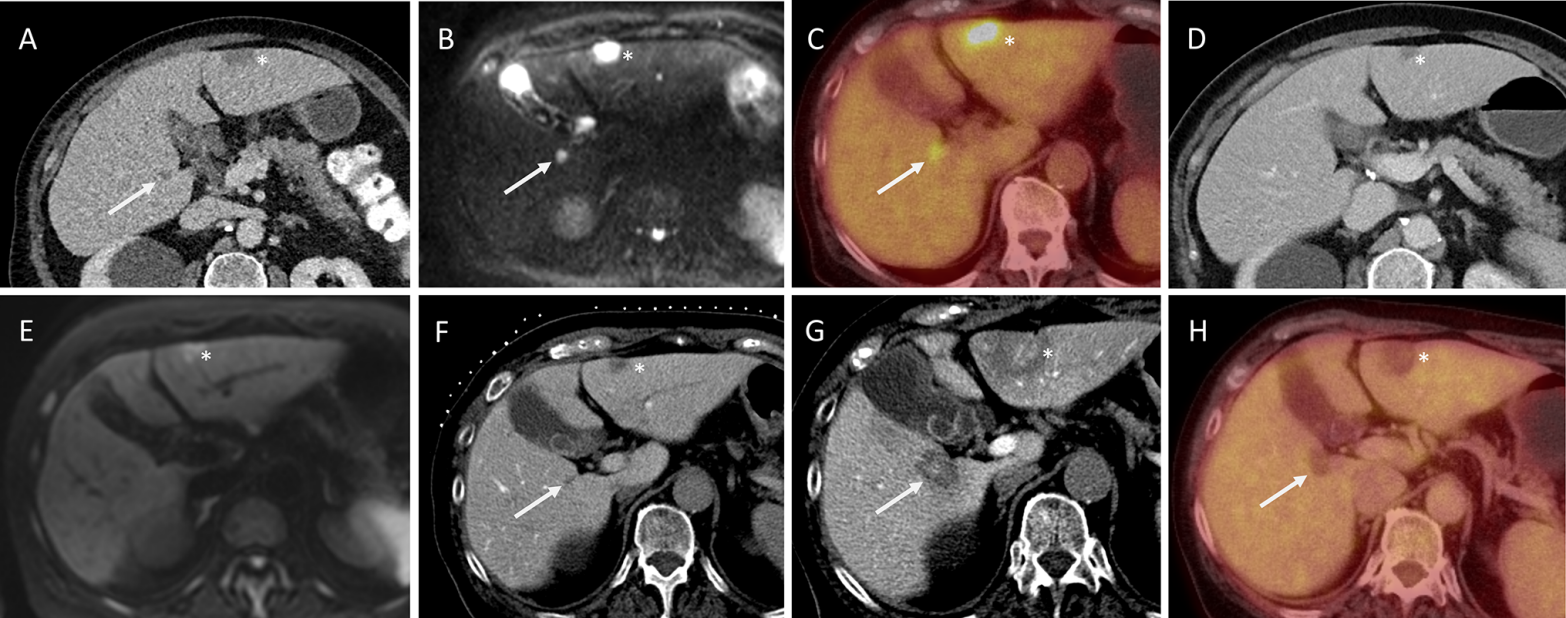
Contrast-enhanced (ce) CT and ceMRI images (**A, B**) and an 18F-FDG PET-CT image (**C**) from a 60-year-old men with two colorectal liver metastases in segment III (*) and segment V (white arrow) from a primary rectal carcinoma. After treatment with chemoradiotherapy for the primary rectal carcinoma, the metastasis in segment V disappeared on both ceCT (**D**) and ceMRI (**E**). On the intraprocedural CT hepatic arteriography (CTHA), both the metastases in segment III as well as the ‘disappeared’ metastasis in segment V were detected (**F**) and treated with percutaneous MWA (**G**) in the setting of the randomized controlled COLLISION trial. Six months after the ablation, there were no signs for local or distant tumour progression on the follow-up 18F-FDG PET-CT (**H**)

A total of 17 CRLM (*n* = 17/357; 4.8%) were not detected on any pre-procedural diagnostic scan and visualized for the first time with CTHA (Fig. 2). The detection led to a change in treatment plan as all tumours were ablated within the same session (*n* = 15/191; 7.9%). The rate of additionally detected CRLM did not differ between the subgroup of patients treated with versus without induction chemotherapy prior to the procedure (*p* = 0.534). The rate of pre-treated patients with additionally detected CRLM was 6/46 (13.0%) versus 9/106 (8.5%) in the group without pre-treatment. In two patients, the detected CRLM actually represented CRLM initially disappeared after induction systemic chemotherapy (Fig. 3).

CTHA failed to visualize six CRLM (overall accuracy 98.3%), all also not detected with ceCT, but detected with ceMRI. In these cases, the area was successfully ablated using ultrasound, real-time fusion imaging with MRI or 18F-FDG PET or using anatomical landmarks.

### Accuracy of ceCT, ceMRI and 18F-FDG PET-CT Without Versus With CTHA

In this study, accuracy of ceCT alone in detecting pre-procedurally known CRLM (diagnosed with ceMRI and/or 18F-FDG PET-CT) was 76.5%. Compared to ceCT alone, CTHA detected 69 additional lesions. The McNemar test showed a statistically significant difference in detecting CRLM, favouring ceCT plus CTHA over ceCT alone (*P* < 0.001).

Accuracy of ceMRI alone was 88.8%. By adding CTHA, an additional 28 pre-procedural diagnosed CRLM (with CT and/ or 18F-FDG PET-CT) were found over ceMRI alone (*P* < 0.001) (Table 2). As mentioned above, six pre-procedurally diagnosed CRLM visible on ceMRI were not identified on CTHA.

**Table 2:** McNemar analysis

Modality	Number of valid cases	Significance
CTHA + CECT VS CECT	332	< 0.001
CTHA + CEMRI VS CEMRI	277	< 0.001
CTHA + 18F-FDG PET-CT VS 18F-FDG PET-CT	240	< 0.001

Accuracy of 18F-FDG PET-CT alone proved to be 85.8%. By adding CTHA, an additional 33 pre-procedural diagnosed (with ceCT and/or ceMRI) CRLM were found. Again, the McNemar analysis showed a statistically significant difference in detecting CRLM, favouring CTHA over 18F-FDG PET-CT alone (*P* < 0.001).

No complications related to the catheter placement were reported. Technical success overall and for concomitantly detected CRLM was 100%. The overall complication rate for the ablative procedures was 15.2% (Common Terminology Criteria for Adverse Events (CTCAE) grades I–II: *n*_s_ = 25/191; 13.1%; CTCAE grade III: *n*_s_ = 4/191; 2.1%). No major and one minor complication (CTCAE grade I: *n*_s_ = 1/17; 5.9%), a small hematoma of the hepatic capsule, was likely related to the ablation of the concomitant CRLM. Median hospital stay was 1 day (range 1–6 days).

### Retrospective Assessment of CTHA After New CRLM in Follow-Up

**Fig. 4:**
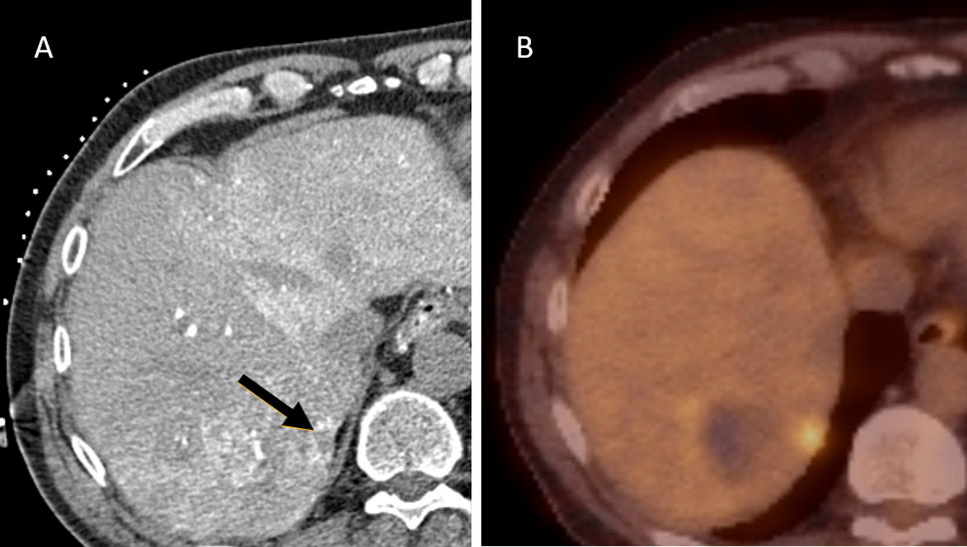
**A** intraprocedural transcatheter CTHA.** B**: follow-up 18F-FDG PET-CT 2,5 months after CTHA-guided ablation. Retrospectively, an enhancing ring lesion was identified in segment VII on the CTHA (arrowheads), which had not been noticed during the microwave ablation

**Fig. 5:**
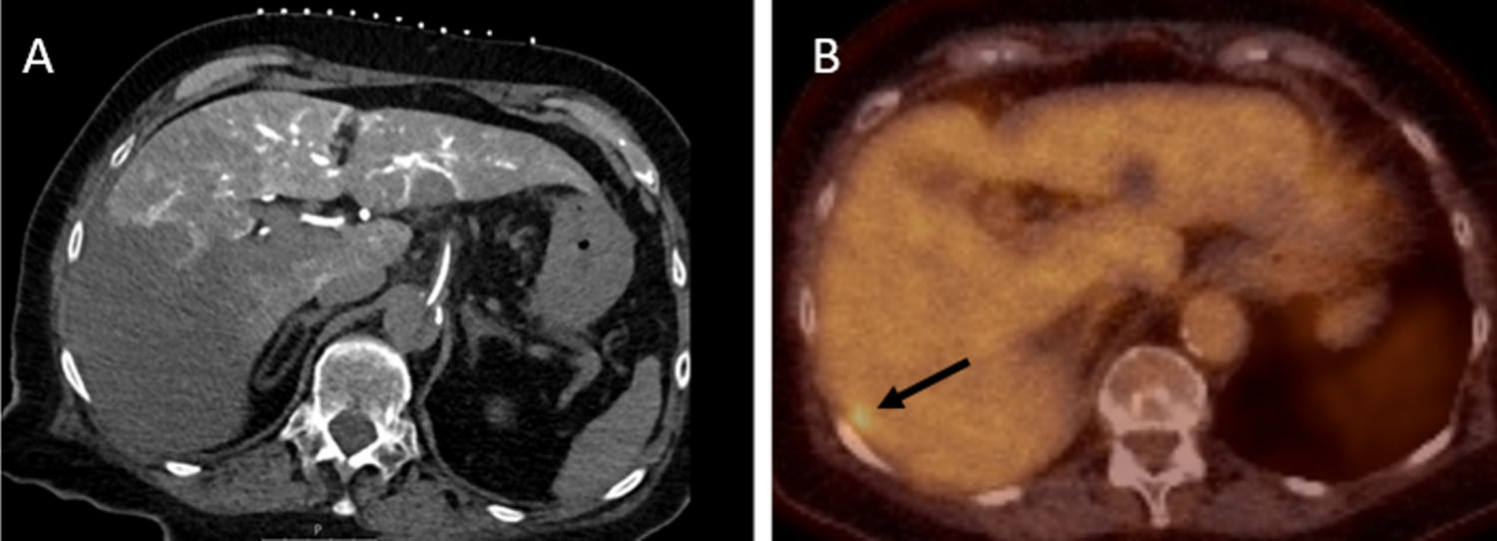
Case with aberrant anatomy: aberrant right hepatic artery from the superior mesenteric artery. This case presents a patient with an aberrant right hepatic artery originating from the superior mesenteric artery, resulting in a CTHA (catheter in the common hepatic artery originating from the celiac trunk) of which a large part of the right hepatic lobe could not be assessed. The follow-up 18F-FDG PET-CT reveals a subcapsular CRLM in segment VII that was subsequently invisible on CTHA, due to the segmented vascular supply

A total of 49 patients who were treated with 62 transcatheter CTHA-guided percutaneous ablations developed 121 new CRLM in follow-up (Table 3). After a blinded assessment of all 62 CTHA scans, additional lesions suspect for CRLM were scored in 24 scans (n_crlm_ = 24/62; 39%). In retrospect, a total of 60 additional tumours were detected on CTHA: 56 true positives (93.3%) and 4 (6.7%) false positives (positive predictive value 93%). In retrospect, 46.3% (n_crlm_ = 56/121) CRLM were already visible on CTHA (Fig. 4). Another 13 CRLM (n_crlm_ = 69/121; 57.0%) were discovered after revealing the follow-up scans and hence unblinding the location where the CRLM would later appear. Ring enhancement was the most frequently reported imaging characteristic of retrospectively identified CRLM (75.0%). Interobserver agreement per CTHA scan was considered substantial (*k* = 0.75). Seven patients had an aberrant anatomy leading to a part of the liver not enhancing with CTHA (Fig. 5).

**Table 3:** Retrospective assessment and tumour characteristics

Retrospectively detected tumours	
Blinded	
No. of CTHA scans with additional tumours (%)	24 (39%)
No. of concomitant true-positive CRLM, N (%)	56 (93.3%)
No. of false-positive lesions scored on CTHA, N (%)	4 (6.7%)
Unblinded	
No. of concomitant true-positive CRLM, N	69
Tumour characteristics (N = 56)	
Enhancing ring, N (%)	42 (75.0%)
Hypodense tumour, N (%)	2 (3.5%)
Hyperdense tumour, N (%)	8 (14.0%)
Mixed attenuation, N (%)	2 (3.5%)
A specific nodule, N (%)	2 (3.5%)

## Discussion

Compared to the conventional cross-sectional imaging modalities (ceCT, ceMRI and 18F-FDG PET-CT), adding CTHA was superior for the detection of CRLM. Furthermore, retrospective CTHA image assessment showed a remarkable number of true-positive CRLM (46.3%), unappreciated during the initial treatment, but detected on follow-up imaging. Taking into account the low number of false positives (6.7%), the favourable safety profile of percutaneous thermal ablation and the substantial interobserver agreement (kappa = 0.75), the immediate ablation of typical ring-enhancing supplementary lesions seems justified and sufficiently validated [16]. Although, in this series, only three patients with rapid disease progression, were not treated with thermal ablation because of the detection of additional CRLM, the depiction of multifocal and scattered disease will prevent some patients from receiving futile ablative procedures.

Compared to other diagnostic modalities, CTHA is considered an effective diagnostic technique with a higher detection rate of both primary and secondary hepatic tumours as is further confirmed by our results [12, 17–21]. Due to its invasive nature and limited influence on the treatment strategy, CTHA is not widely used as a diagnostic tool. However, CTHA-guided percutaneous ablation has an important and increasing role in today's curative-intent treatment options for CRLM, as several studies have demonstrated that CTHA correlates with a reduced risk of local tumour progression (LTP) and increased odds of progression free survival (PFS) [6, 9–12, 22]. This study also suggests that adding CTHA to conventional cross-sectional imaging may help visualize vanished tumours. Furthermore, van Tilborg et al. found transcatheter CTHA to increase operator’s confidence by improving distinction between (vital) residual or recurrent tumour tissue and non-enhancing scar tissue (incomplete ring sign) [11].

To our knowledge, no previous report has assessed the validity to immediately ablate additionally detected lesions suspect for metastases versus to opt for a more conservative wait-and-see approach and treat the lesions at a later stage whenever confirmed on conventional cross-sectional imaging. Arguments in favour of immediate ablation would be to reduce the number of repeat procedures and hence improve quality of life and potentially recurrence-free survival, whereas arguments for the conservative approach would be that these potential benefits may not outweigh the added risks of ablating concomitant potentially false-positive benign liver lesions. Given the very low risk of serious adverse events to ablate small-size CRLM and the high positive predictive value of ring-enhancing lesions found in this study, we suggest to immediately treat the concomitant CRLM as long as the location allows for a safe procedure.

This study has some limitations. First, our inclusion criteria consisted in large part of additionally detected CRLM that were immediately ablated within the same session without histopathological or follow-up confirmation that these actually represented CRLM. For this reason, we were unable to identify false-positive lesions, potentially leading to an overestimation of the accuracy of CTHA. Specificity and positive predictive values could not be analysed due to the missing of false-positive lesions. Therefore, we added a retrospective analysis, where follow-up confirmation was available. However, despite the blinded retrospective assessment, there may still have been confirmation bias due to the fact that our observers knew additional CRLM would appear somewhere in the assessed liver. Though it remains unclear how the intra-procedurally detected and ablated CRLM correlate to the retrospectively found CRLM, it seems likely that the more typical and highly suspect lesions were immediately ablated, which would further strengthen our recommendation to immediately ablate. Furthermore, it should be noted that patients did not all receive identical pre-operative imaging (ceCT, ceMRI and/or 18F-FDG PET-CT). Another limitation is the fact that CTHA is in fact an expansion of ceCT, and the operators were aware of the findings on the pre-procedural ceCT. As a result, the McNemar test has to be interpreted with prudence as CTHA was not assessed as a standalone method. Nonetheless, the high number of additionally detected CRLM on CTHA does render the combination of ceCT and CTHA superior for the detection of CRLM compared to ceCT alone.

The CTHA technique also comes with limitations. Variations in hepatic arterial anatomy such as aberrant right or left hepatic arteries frequently occur [23]. In patients with aberrant anatomy, selective catheterization of the segmental vascular supply makes CTHA of other regions not assessable. Additionally, non-selective catheterization within the aorta or catheter luxation also contributes to a decrease in the quality of the CHTA. In this study, the catheter luxated during transport in 12 out of 273 procedures (4.4%), a weakness that can be overcome by the future implementation of combined angio-CT systems. Additionally, CTHA has well-known pitfalls such as non-tumorous perfusion abnormalities and subsequent formation of pseudo-lesions [18, 24, 25]. Our paper demonstrated no catheter-related complications. However, other studies showed that CTHA comes with a non-negligible risk of vascular complications related to catheter placement, occasionally leading to re-interventions, increased direct costs and radiation dose [12, 19]. Though comparative data are not available, CTHA presumably is more time consuming as the estimated time to place the hepatic artery catheter, to transfer patients to the CT suite, to acquire and assess the CTHA scans and to place a femoral artery closure device (estimated additional time per procedure 20–25 min) questionably outweighs time saved by a superior tumour delineation and a theoretical reduction in required treatments. Several of these shortcomings will likely be solved by the advent and rapid spread of combined angio-CT systems.

In conclusion, this study supports that the hypothesis CTHA is able to detect CRLM not visualized on pre-procedural ceCT, ceMRI and 18F-FDG PET-CT, including CRLM that disappeared after systemic chemotherapy. Due to its invasive character, CTHA is unlikely to replace conventional diagnostic modalities soon. We recommend using transcatheter CTHA in percutaneous ablation procedures (a) to improve visualization and detection of (previously unknown) tumours, (b) to improve the outcome of the percutaneous ablation and (c) to allow for targeted follow-up of indeterminate tumours. The latter requires the interventional radiologist to thoroughly review all liver segments before starting the ablative procedure. Taking into account the low number of false positives and the favourable safety profile of percutaneous thermal ablation, we postulate that immediate ablation of typical ring-enhancing supplementary lesions is sufficiently validated and justified and likely to reduce the number of repeat procedures.
